# Effectiveness of Complementary Therapies in Cancer Patients: A Systematic Review

**DOI:** 10.3390/ijerph18031017

**Published:** 2021-01-24

**Authors:** María Dolores Guerra-Martín, María Sandra Tejedor-Bueno, Matías Correa-Casado

**Affiliations:** 1 Department of Nursing, University of Seville, 41009 Seville, Spain; 2 Faculty of Nursing, Physiotherapy and Podiatry, University of Seville, 41009 Seville, Spain; msandratb@gmail.com; 3 Department of Nursing, Physiotherapy and Medicine, University of Almería, 04120 Almería, Spain; mcc249@ual.es; 4 Public Health Business Agency Hospital de Poniente, 04700 Almería, Spain

**Keywords:** complementary therapies, effectiveness, neoplasms, Randomised Controlled Trial, systematic review, treatment outcome

## Abstract

According to the World Health Organization, cancer is the second leading cause of death in the world. In Spain, about a quarter of a million cases were diagnosed in 2017, and 81% of the Spanish population has used, at least once, some kind of complementary therapy. Said therapies are increasingly being used by cancer patients. The purpose of the study is to analyse the effectiveness of complementary therapies among cancer patients. A systematic peer review was conducted following the PRISMA-ScR guide in four databases (PubMed, CINAHL, Scopus and WOS). The inclusion criteria were Randomised Clinical Trials, published between 2013 and 2018, with a value of 3 or more on the Jadad Scale. The protocol was registered in PROSPERO (CRD42019127593). The study sample amounted to 1845 patients (64.55% women), the most common being breast cancer patients (794), followed by lung cancer patients (341). Fifteen complementary therapies were identified. We found two studies for each of the following: electroacupuncture, phytotherapy, hypnotherapy, guided imagery and progressive muscle relaxation. From the remaining ones, we identified a study on each therapy. The findings reveal some effective complementary therapies: auriculotherapy and acupuncture, laser moxibustion, hypnosis, Ayurveda, electroacupuncture, progressive muscle relaxation and guided imagery, yoga, phytotherapy, music therapy and traditional Chinese medicine. On the other hand, electroacupuncture, laser moxibustion and traditional Chinese medicine presented adverse effects, and kinesiology did not show effectiveness.

## 1. Introduction 

Cancer is the second leading cause of death in the world, especially lung cancer (1.69 million deaths) [1]. Nearly a quarter of a million new cases of cancer were diagnosed in Spain, amounting to 149,000 in men. The most common cancers in men were prostate (22.4%), colorectal (16.6%), lung (15.1%) and urinary bladder (11.7%) cancer; and in women, breast (28.0%), colorectal (16.9%), uterus (6.2%) and lung (6.0%) cancer [2] were the most common.

Traditional or complementary medicine is not usually included in the public health systems of developed countries, although its use is increasing. In this regard, the population who has used complementary therapies (CTs) at least once amounts to 70% in Canada, 42% in the United States, 81% in Spain, 49% in France and 31% in Belgium. Some of the countries that include them in their national health system are Canada, the United Kingdom, Germany and Switzerland. The prevalence of their use is 80% in African countries and 40% in China, where there is an integration between traditional Chinese medicine and acupuncture within the public health system [3,4,5,6,7].

The use of CTs is increasing in the West because of the growing accessibility to health information, as well as an increase in concern about side effects caused by treatments or drugs; however, in Africa and China, this increase is due to the accessibility and affordability of CTs, as well as the strong relationship that traditional medicine has with their belief system and the increasing number of health professionals who practice it [3,4,5,6,7,8,9,10,11].

CTs are a set of knowledge, practices and skills based on indigenous experiences, beliefs and theories from different cultures—whether or not they can be explained—used both for health maintenance and for the prevention, diagnosis, improvement or treatment of physical or mental illnesses [12]. In a study by Borm et al. [10], CTs are defined as “approaches and practices that are typically not part of conventional medical care”. They are considered complementary therapy when these treatments are used in combination with conventional medicine. However, if they are used as a substitute for conventional medicine, they are called alternative therapy. CTs’ classification is wide due to their high diversity and different combinations among them. The United States National Center for Complementary and Integrative Health (NCCIH) classifies them into three large groups. First, we find the group of natural products, which are easily found in pharmacies and herb shops and are usually sold as diet supplements. This group includes products composed of herbs, minerals, vitamins and probiotics. Around 17.7% of adults in the United States used at least one natural product in 2012. Next, we find the mind and body practices group. These practices include yoga, acupuncture, relaxation techniques, therapeutic touch, movement therapies, tai chi, qi gong and hypnotherapy. Among them, yoga, chiropractic treatment, osteopathy, massage therapy and meditation are most frequently used by adults. Lastly, the final group incorporates other complementary methods like traditional Chinese medicine, Ayurveda, naturopathy and homeopathy [10,13,14,15].

In cancer patients, CTs are different from standard medical oncology treatments and include special diets, vitamins, herbs or methods such as acupuncture or massage, among others [16,17]; CTs are mainly oriented to the treatment or prevention of various symptoms caused by cancer treatments or by the disease itself [10]. In addition, the use of CTs causes many patients to feel that they are taking an active role in their health and in the treatment of their illness [18,19,20], as we can also read in the study by Alimujiang et al. [21].

The research question is based on the above. How effective are CTs used to treat cancer patients? The participants were the cancer patients who undergo CTs; the interventions were the various CTs. Comparisons were made between the effectiveness of the various CTs used, and the results were related to the benefits and risks of the various CTs used in cancer patients and the design of the selected randomised clinical trials (RCTs) studied.

The general objective of this study was to update the knowledge regarding the effectiveness of complementary therapies used in cancer patients. To this end, the characteristics of the studies were analysed, the different complementary therapies were described and their effectiveness in cancer patients was compared.

## 2. Materials and Methods 

A qualitative systematic review was carried out, following the Preferred Reporting Items for Systematic Reviews and Meta-Analyses Extension for Scoping Reviews (PRISMA-ScR) guide [22] and different authors [23,24,25]. The review protocol was recorded in the international prospective register of systematic reviews PROSPERO under the number CRD42019127593 (National Institute Health Research, 2019) [26]. This review was carried out using a peer-review process (M.D.G.-M. and M.S.T.-B.), and when there was any discrepancy, a consensus was reached [27].

The following electronic databases were consulted: PubMed, CINAHL, Scopus and Web of Science. Medical Subject Headings descriptors were used. In order for the search strategy to meet the requirements, if reproducible and delimited [25], the same strategy was performed on the different databases: treatment outcome AND neoplasms AND complementary therapies.

The inclusion criteria were as follows: RCTs published in scientific journals addressing the effectiveness of CTs in cancer patients, published in the last 5 years; in English, Spanish or Portuguese; with RCTs scoring 3 or higher on the Jadad Scale [28]. 

There were four stages followed in the selection process of the studies. First, the studies were identified following the established search strategy. Second, an analytical reading of the abstracts, titles and keywords was carried out, and unrelated studies and duplicates were discarded. Third, a thorough and critical reading of the full-text studies was carried out, and those that did not meet the inclusion criteria were discarded. Fourth, studies that did not meet the requirements for methodological assessment according to the Jadad Scale were discarded.

The following data were extracted from the characteristics of each selected study: objectives, study design/methodology, sample/study period/country, complementary therapy/interventions and findings. In addition, the limitations or biases exposed in the various selected studies were collected.

On the one hand, we analysed the sociodemographic variables in terms of the characteristics of the selected CT studies performed on cancer patients, and on the other hand, we analysed the different CTs and their effectiveness. For the analysis of the data, a description of the different variables (frequencies and percentages) that appeared in the different clinical trials of the selected studies was made.

**Figure 1:**
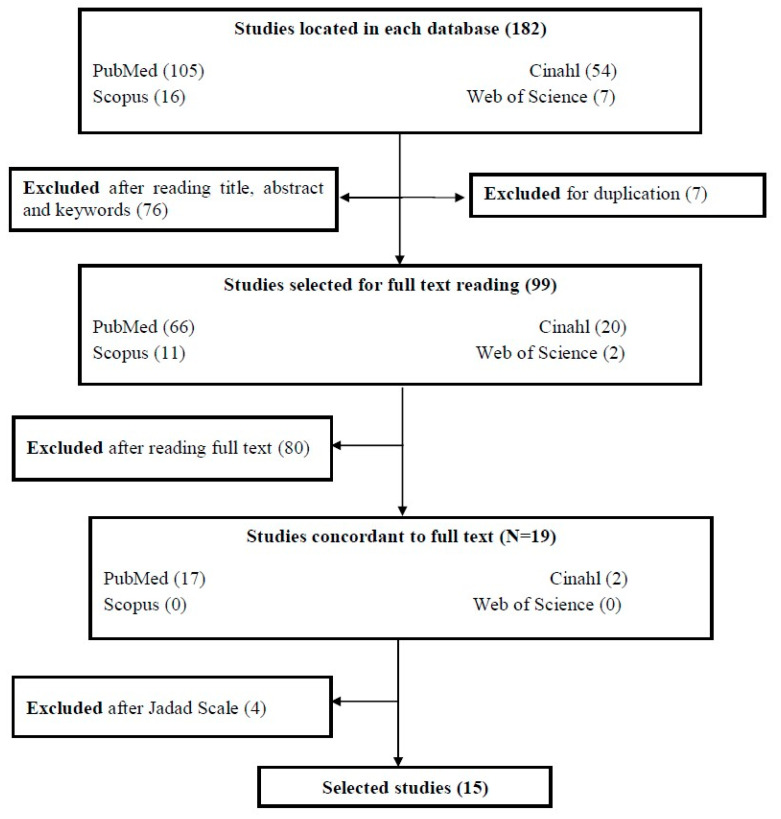
Study selection flowchart.

There were 182 studies identified through the search strategy and, after eliminating duplicates and reading the abstracts and full text, there were 19 studies left. Four of these were excluded after performing the methodological quality assessment using the Jadad Scale, as they obtained a score of less than 3. Fifteen studies were finally selected (Figure 1), of which two studies scored 5 points [29,30], seven scored 4 points [31,32,33,34,35,36,37], and six scored 3 points [38,39,40,41,42,43].

## 3. Results

### 3.1. Characteristics of the Selected Studies 

The study sample included 1845 patients (64.55% women), of whom 794 had breast cancer [31,32,33,34,36,39,40,43], 50 had head and neck cancer [35], 220 had pancreatic cancer [38], 104 had prostate cancer and 104 had breast cancer [36], 341 had lung cancer [41], 142 had liver cancer [37] and 194 had an unspecified type of cancer [29,30,42]. Supplementary Material Table S1 presents the objectives, study design/methodology and sample/data collection/country.

### 3.2. Complementary Therapies 

Regarding the types of CT, 15 therapies were identified. We found a study of each of the following therapies: kinesiology [31], Ayurveda [32], music therapy [39], yoga [40], laser moxibustion [29] and traditional Chinese medicine [30]. We found one study addressing two therapies (acupuncture and auriculotherapy) [41]. We also found two studies of each of the following: electroacupuncture [32,37], phytotherapy [33,38] and hypnotherapy [34,42]. In addition, we found two studies addressing progressive muscle relaxation (PMR) and guided imagery [36,43], and one of them also covered hypnotherapy, acupressure and meditation [43].

### 3.3. Effectiveness of Complementary Therapies 

As for the effectiveness of the various CTs, two studies fail to demonstrate any effectiveness for these therapies: kinesiology [31] and phytotherapy [33]. On the other hand, we found some effective therapies for all the factors studied: hypnosis [34,42], Ayurveda [32], laser moxibustion [29] and auriculotherapy together with acupuncture [41]. We also identified some therapies that are effective on almost all factors: electroacupuncture [42], yoga [40], PMR and guided imagery [36,43], phytotherapy [38] and traditional Chinese medicine [30]. However, there are two therapies that have been shown to be effective on only one factor in the study: electroacupuncture [37] and music therapy [39]. Moreover, some authors identify some safe CTs with no associated adverse events: kinesiology [31], auriculotherapy together with acupuncture [41], PMR and guided imagery [43], hypnosis [34,42], phytotherapy [38], Ayurveda [35] and music therapy [39]. Supplementary Material Table S2 presents CTs, interventions and findings.

### 3.4. Limitations and/or Biases of the Selected Studies 

Table 1 presents the limitations and/or biases of the selected studies.

## 4. Discussion 

In terms of the effectiveness of CTs in cancer patients, there are therapies that show no effect compared to the control group or the therapy to be compared, such as kinesiology [31] or phytotherapy [33]. In contrast, there are therapies, such as Ayurveda [35], hypnosis [34,42], laser moxibustion [29] and auriculotherapy together with acupuncture [41], which present significant results, in terms of effectiveness throughout the follow-up of the RCT, as a solution to resolve symptoms derived from some treatments or from the disease itself. These would be, for acupuncture and auriculotherapy, a decrease in the occurrence of postsurgical constipation [41]; for laser moxibustion, a decrease in cancer-related fatigue [29]; for hypnosis, an improvement in fatigue [34,42], sleep disturbance and pain [42], coinciding with the results of other authors concerning the pain-decrease effect [44]; and for Ayurveda, a decrease in the occurrence of radiodermatitis and an improvement of its symptoms [35].

Regarding electroacupuncture, Xie et al. [37] reported that of the three elements studied (nausea–vomiting, anorexia and the MD Anderson Symptom Inventory (MDASI) scale), only a significant decrease in anorexia was observed. In contrast, Mao et al. [32] obtained better results in their study because they observed improvements in fatigue, anxiety and depression. However, we cannot compare both RCTs as they do not analyse the same elements.

Regarding studies on phytotherapy, the study by McCann et al. [33] mentioned above argues that the intervention carried out did not produce effective results. Although in another study it is shown that there is no clear scientific evidence about mistletoe effectiveness [10], Tröger et al. [38] suggest that phytotherapy with mistletoe therapy has benefits in 13 of the 15 items obtained from the European Organisation for Research and Treatment of Cancer (EORTC) scale, highlighting improvements in pain, fatigue, anorexia and depression. This indicates that not all phytotherapy interventions are effective, but that mistletoe therapy applied in the RCT by Tröger et al. [38], for example, has great benefits for the patient. The effectiveness of these therapies is supported by other authors, such as in the study by Codini et al., where the effectiveness of vitamin C is studied and it is found that this treatment provides broad benefits for cancer patients [45].

Regarding PMR and guided imagery therapy, Charalambous et al. [36] and Stoerkel et al. [43] agree that its use improves fatigue and pain but contradict each other in terms of anxiety, which is found to decrease in the RCT by Stoerkel et al. [43] and has nonsignificant results in the RCT by Charalambous et al. [36]. This result is due to the fact that PMR and guided imagery were not the only therapies used in the study by Stoerkel et al. [43], as other therapies such as self-hypnosis, acupressure and meditation also played a role, making the set of different therapies more effective, as we can observe in the studies by Araujo et al. [46] and Tacón et al. [47], where it is highlighted that meditation yields positive effects like lower anxiety levels in patients with cancer, in addition to other factors, as the study by Zhang et al. also considers [48].

Studies of traditional Chinese medicine [30], music therapy [39] and yoga [40] also fail to show significant effects on all study factors. In the first study [30], there is significant improvement in chest discomfort, fullness and distension and shortness of breath, but not for palpitations and pain. Moreover, other authors consider the use of traditional Chinese medicine in the decrease of side effects caused by cancer treatments such as chemotherapy or radiotherapy [49]. In the second [39], there is a greater reduction in perioperative anxiety in both intervention groups, but not in the other factors to be studied. However, in the yoga RCT [40], they are not present in all of them, but only one of the components studied does not give a significant positive result. On the other hand, in the systematic review by Behzadmehr et al. [44], yoga and music therapy are effective in the reduction of pain, a factor that was not highlighted in the studies about music therapy [39] and yoga [40] of this review. Similar results were found in the study by Gosain et al. [50], where they affirm that, in addition to yoga being effective, this therapy also increases tolerance to side effects of chemotherapy.

Thus, it is observed that therapies such as electroacupuncture [32,37], phytotherapy [38], PMR and guided imagery [36,43], traditional Chinese medicine [30], music therapy [39] and yoga [40] are effective in some of the items studied, and they are mostly beneficial.

Regarding the risks or adverse effects of CTs in cancer patients, the RCTs on electroacupuncture [37], laser moxibustion [29] and traditional Chinese medicine [30] described side effects, although they are listed as mild and rapidly resolving symptoms. Depending on the therapy, these are mild erythema, with 3 cases in 39 patients using laser moxibustion [29]; mild tingling and redness, with 2 cases in 79 patients using electroacupuncture [37]; and skin irritation and allergic reaction, with 4 cases in 36 patients using traditional Chinese medicine [30]. However, in relation to traditional Chinese medicine, the study of Wang et al. affirms that there is little evidence to validate the therapy’s safety [51].

On the other hand, we identified nine therapies in different studies that are considered to be safe, i.e., that do not pose any adverse effects. These are kinesiology [25]; auriculotherapy together with acupuncture [35]; hypnosis [28,36]; phytotherapy [32]; Ayurveda [29]; music therapy [33]; and PMR and guided imagery together with self-hypnosis, acupressure and meditation [37], coinciding with the results of other authors such as Araujo et al. [46] and Gosain et al. [50], who show that meditation is a safe intervention. 

Despite this fact, as it is observed in the studies by Thomford et al. [52] and Thomford et al. [53], in relation to phytotherapy, CTs can cause interactions with conventional medicine, some with fatal clinical outcomes. However, we found that some studies did not mention the risks or adverse effects in the use of certain therapies, such as electroacupuncture [32], PMR and guided imagery [36], yoga [40] and phytotherapy [33].

As for limitations, the first is related to the impossibility of retrieving all existing information on the topic of study, since searches have been restricted to the last 5 years and by language. We tried to alleviate this language limitation by including English, Spanish and Portuguese, since most studies are published in English. The second limitation is the heterogeneity of the studies in terms of samples, study periods, interventions and results, although some authors believe that if the data are treated rigorously and methodically, as has been done in this review, they are a beneficial contributing source to the research [54].

## 5. Conclusions

The findings reveal some effective CTs: auriculotherapy and acupuncture, laser moxibustion, hypnosis, Ayurveda, electroacupuncture, progressive muscle relaxation and guided imagery, yoga, phytotherapy, music therapy and traditional Chinese medicine. However, other CTs—such as electroacupuncture, laser moxibustion and traditional Chinese medicine—presented adverse effects, and kinesiology did not show effectiveness. 

These results may be useful when making decisions in clinical management, although it would be advisable to continue research on this subject, carrying out more RCTs that can provide greater certainty about the influence of sample size, follow-up periods and/or other variables on the results. All of this should be performed in order to evaluate the scientific evidence and to corroborate or contrast the results obtained in this review, so as to obtain greater insight into the effectiveness of CTs for cancer patients.

## Figures and Tables

**Table 1:** Limitations and/or biases of the selected studies.

Authors/Year	Limitations and/or Biases of the Studies
Smykla et al., 2013 [31]	The sample size is too small to show significant effects.
Mao et al., 2014 [32]	The study was not able to detect a statistically significant difference between electroacupuncture and simulated electroacupuncture. Follow-up period too short to see differences between electroacupuncture and simulated electroacupuncture. The simulated electroacupuncture group cannot function as a physiologically inert placebo group due to tactile stimulation.
McCann et al., 2014 [34]	The sample size is too small to show significant effects. The time between the biopsy and the resection is limited to a short period of time.
Montgomery et al., 2014 [33]	Relatively small sample. Lack of a professional care control group. The fatigue measurement ended at the end of the radiation therapy and prevented us from knowing if there is any benefit after the intervention.
Palatty et al., 2014 [35]	It is not a double-blind study: the patients knew the prophylactic treatment assigned. Small sample size.
Tröger et al., 2014 [38]	No record.
Palmer et al., 2015 [39]	Finding the ideal time to introduce music therapy intervention in a fast-paced preoperative environment was a significant logistical challenge. Due to the nature of the treatment, there was no blinding. Doubts about whether the effect was due to the additional presence of an attentive professional. Participants received additional personalised attention: preoperative phone call, special welcome and anxiety assessments.
Charalambous et al., 2016 [36]	Inability to perform a double-blind RCT due to the type of intervention: difficulty in controlling the placebo effect. It is not possible to know for certain whether patients always performed the protocol in its entirety. The researchers do not know if patients, during the unsupervised sessions, chose an external environment without stimuli or had to interrupt the session, as any interruption in the protocol could have impacted its effectiveness.
Loudon et al., 2016 [40]	Lack of evidence of familiarity prior to data collection. Differences between groups of certain variables in the baseline. Small sample size.
Mao et al., 2016 [29]	Phase 2 study with a limited sample size and a short follow-up period. Drop-out rate of 20%, due to worsening clinical conditions due to active cancer treatment. The lack of a longer follow-up period makes it impossible to assess the long-term effects of this treatment. The lack of a usual care group did not allow the estimation of the overall effect of laser moxibustion on fatigue. Very specific study population.
Feize et al., 2017 [30]	Sample size too small (n = 36 for each arm). Inclusion of subjects needing thoracentesis or drainage in the study. There was no consensus on the grading of pleural effusion sonograms. 14-day study observation period. There was no record of the stage of the cancer, which could help identify the confounders caused by the remission of cancer.
Li et al., 2017 [41]	No record.
Mendoza et al., 2017 [42]	Only one clinician, who was not blind to the hypotheses, provided both treatments to all participants. There were relatively few men in the sample. It was not possible to assess the relative contribution of each treatment to the overall benefits observed. Expectations for treatments were not measured, the potential role of other mechanisms that might explain the outcome was not evaluated.
Xie et al., 2017 [37]	No record.
Stoerkel et al., 2018 [43]	Lengthy administrative processes for approval of the study by the Military Institutional Review Board. Adding a second centre for increased enrolment, where many patients from these centres were referred to other facilities. Start of the intervention at the time of diagnosis, which is potentially useful for emotional distress, but not a priority for many patients. The intervention of the MP3 player used was surpassed by technological advances in mobile applications.

RCT = Randomised clinical trial.

## Data Availability

The review protocol was recorded in the international prospective register of systematic reviews PROSPERO under the number CRD42019127593. Available online: https://www.crd.york.ac.uk/prospero/display_record.php?RecordID=127593.

## Supplementary Materials

The following are available online at https://www.mdpi.com/1660-4601/18/3/1017/s1: Table S1: Characteristics of the selected studies. Table S2: Complementary therapy/interventions and findings.
