# Supplementary material for: Effectiveness of Complementary Therapies in Cancer Patients: A Systematic Review
Source: Int J Environ Res Public Health. 2021 Jan 24;18(3):1017. doi: 10.3390/ijerph18031017 (PMC7908482; doi:10.3390/ijerph18031017)
Supplement: Supplementary file 1 [file ijerph-18-01017-s001.pdf]

Supplementary Material Table S1. Characteristics of the selected studies.

Authors/<br>Year	Aim(s)	Design/<br>Study Methodology	Sample/Data<br>Collection/Country
Smykla et al., 2013 [31]	To evaluate the effectiveness of kinesiology tapes in treating lymphedema related to breast cancer.	Design: RCT*<br>Sampling: Simple random<br>Blinding: Yes<br>Recruitment: Provita Clinic in Zory and Limf-Med Hospital in Chorzów	Sample: 75 patients<br>KG†: 20<br>GQK‡: 22<br>GMC§: 23<br>Data collection: December 2012–August 2013<br>Country: Poland
Mao et al., 2014 [32]	To assess the effect of electroacupuncture on fatigue, sleep and anxiety in breast cancer patients experiencing joint pain related to aromatase inhibitors.	Design: RCT*<br>Sampling: Simple random<br>Blinding: Yes<br>Recruitment: Abramson Cancer Center at the University of Pennsylvania Hospital	Sample: 67 patients<br>CG‖: 23<br>GEA¶: 22<br>GSEA **: 22<br>Data collection: September 2009–May 2012<br>Country: USA
McCann et al., 2014 [33]	To assess the effects of flaxseed and the aromatase inhibitor, anastrozole, and possible interactions between them, on serum steroid hormones (androsterone, testosterone, and DHEA††) and tumour-related characteristics linked to long-term survival. To research the possible interaction between F and A in these biomarkers (oestrogen receptor β).	Design: RCT*<br>Sampling: Simple random<br>Blinding: Yes<br>Recruitment: Roswell Park Cancer Institute	Sample: 24 patients<br>GF‡‡: 6<br>GFA§§: 6<br>GA‖‖: 7<br>GP¶¶: 5<br>Data collection: November 2007–April 2014<br>Country: USA
Montgomery et al., 2014 [34]	To examine the efficacy of cognitive behavioural therapy plus hypnosis in managing fatigue in breast cancer patients undergoing radiation therapy.	Design: RCT*<br>Sampling:<br>Simple random<br>Blinding:<br>Yes<br>Recruitment:<br>Mount Sinai Medical Center	Sample:<br>200 patients<br>GCBTH***: 100<br>CG‖: 100<br>Data collection: January 2006–July 2011<br>Country: USA
Palatty et al., 2014 [35]	To evaluate the effectiveness of a cream containing turmeric wood oil and sandalwood (Vicco turmeric cream) on radiodermatitis in head and neck cancer patients undergoing radiation therapy.	Design: RCT*<br>Sampling: Simple random<br>Blinding: Yes<br>Recruitment: Department of Radiation Oncology of the Father Muller Medical College	Sample: 50 patients<br>CG‖: 25<br>GVTC†††: 25<br>Data collection: September 2010–October 2011<br>Country: India
Tröger et al., 2014 [38]	To study the effectiveness of mistletoe monotherapy on survival and quality of life in patients with metastatic or locally advanced pancreatic carcinoma.	Design: RCT*<br>Sampling: Simple random<br>Blinding: No<br>Recruitment: Oncology consultations at Clinical Centres in Serbia	Sample:<br>220 patients<br>CG‖: 110<br>GI‡‡‡: 110<br>Data collection: January 2009–December 2010<br>Country: Serbia
Palmer et al., 2015 [39]	To determine if music therapy affects anxiety levels, anaesthesia requirements, recovery time, and satisfaction in patients who will undergo outpatient breast surgery.	Design: RCT*<br>Sampling: Simple random<br>Blinding: No<br>Recruitment: University Hospitals Case Medical Center and Richmond	Sample: 201 patients<br>CG‖: 65<br>GLM§§§: 68<br>GRM‖‖‖: 45<br>Data collection: 2012–2014

		University Medical Center	Country: USA
Charalambous et al., 2016 [36]	To determine whether guided imagery and progressive muscle relaxation techniques are effective in the treatment of pain, fatigue, nausea, vomiting, anxiety and depression in patients with prostate or breast cancer.	Design: RCT*<br>Sampling: Simple random<br>Blinding: Yes<br>Recruitment:<br>Bank of Cyprus Oncology Centre	Sample:<br>208 patients<br>CG||: 104<br>GI‡‡‡: 104<br>Data collection: April 2010–October 2011<br>Country: Cyprus
Loudon et al., 2016 [40]	To assess the effect of an 8-week yoga intervention on the shoulders and spines of women with lymphedema related to breast cancer.	Design: RCT*<br>Sampling: Simple random<br>Blinding: No<br>Recruitment: Through health professionals, community groups and local radio and newspaper media	Sample: 23 patients<br>CG||: 12<br>GI‡‡‡: 11<br>Data collection: February 2011–April 2011<br>Country: Australia
Mao et al., 2016 [29]	To assess the preliminary effectiveness and safety of 10.6 μm infrared laser moxibustion for cancer-related fatigue.	Design: RCT*<br>Sampling: Simple random<br>Blinding: Yes<br>Yueyang Hospital Recruitment	Sample: 78 patients<br>CG||: 39<br>GI‡‡‡: 39<br>Data collection: September 2011–February 2014<br>Country: China
Feize et al., 2017 [30]	To observe the safety and effectiveness of Kang'ai Xiaoshui Ointment (herbal ointment) on malignant pleural effusion.	Design: RCT*<br>Sampling: Simple random<br>Blinding: Yes<br>Recruitment: China-Japan Friendship Hospital, Third Affiliated Hospital of the University of Chinese Medicine and Hospital of Traditional Chinese Medicine	Sample: 72 patients<br>CG||: 36<br>GI‡‡‡: 36<br>Data collection: 14 days (no year specified)<br>Country: China
Li et al., 2017 [41]	To assess the effect of ear point treatment combined with the application of acupuncture points in patients with constipation after lung cancer surgery.	Design: RCT*<br>Sampling: Simple random<br>Blinding: No<br>Recruitment: Department of Thoracic Surgery, Medical University Cancer Hospital	Sample:<br>341 patients<br>CG||: 167<br>GI‡‡‡: 174<br>Data collection: March 2014–May 2015<br>Country: China
Mendoza et al., 2017 [42]	To evaluate the effectiveness of VMWH¶¶¶ when combined with CBT**** in a sample of active cancer patients or survivors after treatment who report bothersome pain, fatigue or sleep problems.	Design: RCT*<br>Sampling: Simple random<br>Blinding: Yes<br>Recruitment: Previous study participants, Seattle cancer survivors and patients of the Women's Wellness Clinic at Seattle Cancer Care Alliance	Sample: 44 patients<br>GVMWH-CBT††††:22<br>GEC‡‡‡‡:22<br>Data collection: 4 months (2011–2012)<br>Country: USA
Xie et al., 2017 [37]	To study the clinical effects of transcutaneous electrical stimulation of the acupuncture point on gastrointestinal complaints (nausea, vomiting, anorexia) following transcatheter arterial chemoembolization.	Design: RCT*<br>Sampling: Simple random<br>Blinding: Yes<br>Recruitment: Department of Integrative Oncology of the<br>Fudan University Shanghai Cancer Centre	Sample: 142 patients<br>CG||: 72<br>GI‡‡‡: 70<br>Data collection: June 2013–December 2014<br>Country: China

Stoerkel et al., 2018 [43]	To determine whether self-care tools with guided mind–body techniques, delivered before and after the operation, would be more effective than usual treatment in reducing anxiety and symptoms associated with surgery in women with newly diagnosed breast cancer.	Design: RCT[*]<br>Sampling: Simple random<br>Blinding: No<br>Recruitment: Brooke Army Medical Center and Carl R. Darnall Army Medical Center	Sample: 100 patients<br>CG[‖]: 51<br>GI[‡‡‡]: 49<br>Data collection: July 2017–February 2014<br>Country: USA

*RCT= Randomised clinical trial; †KG= Kinesiology group; ‡GQK= Quasi-kinesiology group; §GMC= Multiple compression group; ‖CG= Control group; ¶GEA= Electroacupuncture group; **GSEA= Simulated electroacupuncture group; ††DHEA= Dehydroepiandrosterone; ‡‡GF= Flaxseed group; §§GFA= Flaxseed-anastrozole group; ‖‖GA= Anastrozole group; ¶¶GP= Placebo group; ***GCBTH= Cognitive behavioural therapy-hypnosis group; †††GVTC= Vicco turmeric cream group; ‡‡‡GI= Intervention group; §§§GLM= Live music therapy group; ‖‖‖GRM= Recorded music therapy group; ¶¶¶VMWH= Valencia model of waking hypnosis; ****CBT= Cognitive behavioural therapy; ††††GVMWH-CBT= Valencia model of waking hypnosis with cognitive behavioural therapy; ‡‡‡‡GEC= Educational control group.

Supplementary Material Table S2. Complementary therapy/interventions and findings.

Authors/Year	Complementary Therapy/ Interventions	Findings
Smykla et al., 2013 [31]	Kinesiology Intervention: Standard therapy (skin care, 45-min pneumatic compression therapy, 1-h manual lymphatic drainage and application of short stretch multilayer bandages) 3 times a week.<br>In the case of KG[*] and GQK[‡], they are kinesiology or quasi-kinesiology tapes instead of bandages.<br>Aimed at: Women with breast cancer and stage 2–3 unilateral lymphedema.	The average volume of affected limbs in KG[*] was 9414.01 cm^3, and it decreased after treatment to 8051.15 cm^3 (p=0.002); in GQK[‡] it was 9621.33 cm^3, and it decreased to 8041.02 cm^3 (p=0.002); in GMC[‡] it was 10,089.41 cm^3, and fell to 5021.22 cm^3 (p=0.000001) after treatment. The most significant decrease in oedema was in GMC[‡]. They conclude that kinesiology tape cannot replace the multiple compression bandage and should not currently be an alternative option for patients with lymphedema related to breast cancer.
Mao et al., 2014 [32]	Electroacupuncture<br>Intervention: GEA[§]: Electroacupuncture intervention in 10 sessions.<br>GSEA[‖]: Simulated electroacupuncture intervention in 10 sessions.<br>Aimed at: Women with stage 1–3 breast cancer with aromatase-inhibitor-related joint pain.	Compared to CG[¶], electroacupuncture caused significant improvement in fatigue (p=0.0095), anxiety (p=0.044) and depression (p=0.015) and non-significant improvement in sleep disturbance (p=0.058). In contrast, simulated electroacupuncture did not cause a significant reduction in fatigue and anxiety symptoms, but it did cause a significant improvement in depression compared to CG[¶] (p=0.0088). They conclude that electroacupuncture caused a significant improvement in fatigue, anxiety and depression, while simulated electroacupuncture improved only depression compared to CG[¶].
Montgomery et al., 2014 [34]	Hypnotherapy<br>Intervention: Cognitive-behavioural therapy sessions plus 15-min hypnosis.<br>Aimed at: Breast cancer patients undergoing radiation therapy.	GCBTH[**] has significantly lower levels of fatigue at the end of radiation therapy, at 4 weeks' follow-up, and at 6 months' follow-up. They conclude that the therapy performed in the GCBTH[**] is effective in controlling fatigue related to breast cancer radiation therapy. It is also a non-invasive therapy, without adverse effects and with persistent beneficial effects over time.
McCann et al., 2014 [33]	Phytotherapy<br>Intervention: GF[††]: Taking 25 mg of flaxseed and 1 placebo pill per day.<br>GFA[‡‡]: Taking 25 mg of flaxseed and 1 mg of anastrozole pill per day.<br>GP[§§]: Taking 1 placebo pill per day.<br>GA[‖‖] Taking 1 anastrozole pill, 1 mg, per day. Aimed at: Postmenopausal women diagnosed with oestrogen receptor-positive breast cancer who were to receive surgery.	Compared to GP[§§], no effect was observed in GF[††], GA[‖‖] or GFA[‡‡] on the expression of growth-related biomarkers or oestrogen receptor β expression. There were negative results for androstenedione in GFA[‡‡] compared to GP[§§]. For DHEA[¶¶] the serum levels were negative in the GA[‖‖]. They conclude that no effects of flaxseed on aromatase inhibitor activity are observed with respect to selected characteristics of breast tumours, growth hormone or serum steroid hormone levels.
Palatty et al., 2014 [35]	Ayurveda<br>Intervention: Application of ointment (Johnson Baby Oil 2 mL on CG[¶] and GVTC[***] 2 mg) five times a day: 2 h before, immediately after, 2 h after and 4 and 6 h after radiation therapy.<br>Aimed at: Head and neck cancer patients undergoing radiation therapy.	The significant reduction in degrees of dermatitis in GVTC[***] occurs at all time points, including 2 weeks after radiation therapy (p<0.015 to p<0.001). The occurrence of grade 3 dermatitis is less in GVTC[***] and is statistically significant (p <0.01). In the follow-up after 2 weeks of the completion of radiation therapy, a reduced degree of radiodermatitis in GVTC[***] was also observed, and it was significant (p=0.015). No adverse events occurred. They conclude that Vicco turmeric cream is effective in preventing radiodermatitis and reducing grade 3 dermatitis.

Table S2 (continued). Complementary therapy/interventions and findings.

Authors/Year	Complementary Therapy / Interventions	Findings
Tröger et al., 2014 [38]	Phytotherapy<br>Intervention: Subcutaneous injection of *Viscum album* (L.) extract.<br>Aimed at: Patients with advanced pancreatic cancer.	People treated with mistletoe scored better on the 6 scales of the functioning area of the EORTC[†††] scale and on 7 out of 9 of the scales of symptoms, including pain, fatigue, loss of appetite and insomnia. No side effects were observed with mistletoe treatment. They conclude that mistletoe treatment significantly improves quality of life and survival compared to usual care.
Palmer et al., 2015 [39]	Music therapy<br>Intervention: To listen to music recorded on an MP3 player in the operating room (GRM[‡‡‡]).<br>To listen to live music in the operating room (GLM[§§§]).<br>Aimed at: Women undergoing outpatient surgery for the diagnosis or treatment of breast cancer.	The GLM[§§§] and GRM[‡‡‡] did not differ significantly from CG[¶] in the amount of propofol needed to achieve moderate sedation. Compared to the CG[¶], both the GLM[§§§] and GRM[‡‡‡] had greater reductions (p <0.001) in preoperative anxiety scores. The GLM[§§§] and GRM[‡‡‡] did not differ from the CG group with respect to recovery time; however, the GLM[§§§] had a shorter recovery time compared to GRM[‡‡‡] (a difference of 12.4 min). The satisfaction scores for the GLM[§§§] and GRM[‡‡‡] did not differ from those of the CG[¶]. They conclude that the therapy helps to control preoperative anxiety in a pleasant, safe, effective and time-efficient manner.
Charalambous et al., 2016 [36]	PMR[ǁǁǁ] and guided imagery<br>Intervention:<br>4 sessions of guided imagery and PMR[ǁǁǁ] (breathing and muscle relaxation exercises)<br>Aimed at:<br>Patients with prostate or breast cancer.	Patients in the GI[¶¶¶] experienced lower levels of fatigue (p<0, 0225) and pain (p=0.0003) compared to those in the CG[¶] and improved perceived health-related quality of life (p<0.0001). Nausea, vomiting and gagging occurred significantly less frequently in the GI[¶¶¶] when compared to the CG[¶]. More patients in the CG[¶] (pre: n=33 post: n=47) were moderately depressed compared to those in the GI[¶¶¶] (pre: n=35 post: n=15). There was no significant improvement in anxiety. They conclude that mind–body guided techniques are effective in the joint treatment of chemotherapy-related symptoms.
Loudon et al., 2016 [40]	Yoga<br>Intervention: A weekly 90-min guided yoga class for 8 weeks and a daily 45-min practice yoga session on DVD.<br>Aimed at: Women with lymphedema related to breast cancer.	The GI[¶¶¶] had an improvement in lumbopelvic posture in week 8, as indicated by the reduction in pelvic obliquity compared to the CG[¶]. Strength in shoulder abduction was significantly increased after yoga intervention in both the affected and unaffected arm. There were no significant changes between the groups in the range-of-motion measures. They conclude that the performance of yoga provides benefits in posture and strength in these patients.
Mao et al., 2016 [29]	Laser moxibustion<br>Intervention: Normal (GI[¶¶¶]) or simulated (CG[¶]) infrared laser on acupuncture point and routine cancer treatment.<br>Aimed at: Patients with cancer-related fatigue.	Individuals in the GI[¶¶¶] had significantly less fatigue than those in the CG[¶] (3.01 vs 4.40; P=0.002).<br>The improvement in fatigue persisted into the 8th week, favouring the GI[¶¶¶] (3.03 vs 4.26; P=0.006). Laser moxibustion was safe and there were only 3 cases of mild local erythema, which were resolved. They conclude that the therapy is safe and effective for cancer-related fatigue.

Table S2 (continued). Complementary therapy/interventions and findings.

Authors/Year	Complementary Therapy/Interventions	Findings
Feize et al., 2017 [30]	Traditional Chinese medicine<br>Intervention: To apply 10 g Kang'ai Xiaoshui Ointment or placebo to the side wall of the chest on the side of the effusion and cover with a dressing for 8 h a day for 2 weeks.<br>Aimed at: Patients with confirmed malignant tumour by pathology or cytology and detected pleural effusion.	The GI¶¶¶ showed statistical significance in improvement of chest discomfort (p=0.003), fullness and distension (p=0.042), and dyspnea (p<0.001), but there was no statistical significance in palpitation (p=0.237) and pain (p=0.063), while the placebo group showed no statistical significance in any of the five symptoms. The main treatment-related adverse event was skin irritation and allergy, 4 in the GI¶¶¶ and 5 in the GP§§. They conclude that therapy has the potential to reduce malignant pleural effusion and decrease dyspnoea.
Li et al., 2017 [41]	Auriculotherapy and acupuncture<br>Intervention: In the GI¶¶¶ session, 1 week before the intervention, for the application of the auriculotherapy and acupuncture points and teaching of the procedures to follow until discharge.<br>Aimed at: Patients with a diagnosis of lung cancer and upcoming pulmonary lobectomy.	The incidence of constipation in the CG¶ was higher than in the GI¶¶¶ (p <0.001). There was less constipation in older patients (≥60) compared to younger patients (<60) in the GI¶¶¶ (p<0.001). The stool characteristics of the GI¶¶¶ are better than those of the CG¶ by the range-sum test (p=0.047). There are no associated adverse events. They conclude that the application of both therapies together with the usual measures serves to effectively and safely reduce the incidence of postoperative constipation.
Mendoza et al., 2017 [42]	Hypnotherapy<br>Intervention: Four 1-h treatment sessions that combined self-hypnosis training with CBT****.<br>Aimed at: Patients who report bothersome pain, fatigue or sleep problems and are in active cancer treatment or survivors after treatment.	They conclude that the GVMWH-CBT†††† intervention produces clinically significant improvements in pain, fatigue and sleep problems, compared to the educational control intervention. No adverse effects were reported. Benefits are maintained for at least 3 months.
Xie et al., 2017 [37]	Electroacupuncture<br>Intervention:<br>CG¶: Tropisetron 6 mg treatment before TACE‡‡‡‡<br>GI¶¶¶: TENS§§§§ combined with tropisetron 6 mg before TACE‡‡‡‡.<br>Aimed at: Patients with primary or metastatic liver cancer.	In both groups, the differences in rates of occurrence and severity of nausea and vomiting after TACE‡‡‡‡ were not significant. As of day two of TACE‡‡‡‡, anorexia scores were significantly lower in the GI¶¶¶ than in the CG¶ and continued to decline with treatment. On days 0, 1 and 2, the mean MDASI***** scores for GI¶¶¶ were slightly lower than those for CG¶, but the differences were not statistically significant. There were no serious adverse events, only two incidents of mild tingling and redness. They conclude that TENS§§§§ is a safe and effective therapy to use as a complement to relieve gastrointestinal discomfort after chemotherapy.

Table S2 (continued). Complementary therapy/interventions and findings.

Authors/Year	Complementary Therapy/ Interventions	Findings
Stoerkel et al., 2018 [43]	PMR‖‖‖, meditation, guided imagery, suggestions for self-hypnosis and acupressure Intervention: CG¶: Usual preoperative medical care.<br>GI¶¶¶: Teaching the use of self-care tools, MP3 and acupressure wristbands. Use of the material at least once during the preoperative and postoperative periods of 2 weeks. Use of acupressure wrist bands during surgery.<br>Aimed at: Adult women recently diagnosed with nonmetastatic breast cancer and with initial surgical treatment.	Compared to CG¶, there are significant differences in the interference of pain, fatigue and anxiety in GI¶¶¶ from baseline to follow-up. In the immediate perioperative period, GI¶¶¶ had a minor increase in postoperative pain as measured by DVPRS†††††. Satisfaction with social roles and activities decreased significantly in the CG¶ and stabilised in the GI¶¶¶. There were no adverse events. They conclude that the use of SCTs in the perioperative period decreases pain perceptions, fatigue and inflammatory cytokine secretion (anxiety).

*KG= Kinesiology group; †GQK= Quasi-kinesiology group; ‡GMC= Multiple compression group; §GEA= Electroacupuncture group; ‖GSEA= Simulated electroacupuncture group; ¶CG= Control group; **GCBTH= Cognitive behavioural therapy-hypnosis group; ††GF= Flaxseed group; ‡‡GFA= Flaxseed-anastrozole group; §§GP= Placebo group; ‖‖GA= Anastrozole group; ¶¶DHEA= Dehydroepiandrosterone; ***GVTC= Vicco turmeric cream group; †††EORTC= European Organisation for Research and Treatment of Cancer; ‡‡‡GMG= Recorded music therapy group; §§§GMD= Live music therapy group; ‖‖‖PMR= Progressive muscle relaxation; ¶¶¶GI= Intervention group; ****CBT= Cognitive-behavioural therapy; ††††GVMWH-CBT= Valencia model of waking hypnosis with cognitive-behavioural therapy; ‡‡‡‡TACE= Transcatheter arterial chemoembolization; §§§§TENS= Transcutaneous electrical stimulation; *****MDASI= MD Anderson Symptom Inventory; †††††DVPRS= Defense and Veterans Pain Rating Scale.
